# Complement and autoantibody levels under anifrolumab therapy in SLE: implications for clinical practice

**DOI:** 10.3389/fimmu.2026.1737281

**Published:** 2026-03-11

**Authors:** Jan-Gerd Rademacher, Björn Tampe, Peter Korsten

**Affiliations:** 1 Department of Nephrology and Rheumatology, University Medical Center Göttingen, Göttingen, Germany; 2 Department of Rheumatology and Clinical Immunology, St. Josef-Stift Sendenhorst, Sendenhorst, Germany

**Keywords:** antibodies, complement, interferon, SLEDAI, systemic lupus erythematosus

## Abstract

**Introduction:**

Anifrolumab (ANI), a type I interferon receptor antagonist, has demonstrated clinical efficacy in systemic lupus erythematosus (SLE). However, its effects on serological markers commonly used to assess disease activity in clinical practice remain uncertain. This study evaluated changes in complement and autoantibody levels in SLE patients treated with ANI under routine care conditions.

**Methods:**

We performed a single-center retrospective analysis of SLE patients receiving ≥3 ANI infusions over a 12-month period. Clinical and serological data, including complement (C3c, C4), anti-double-stranded DNA (anti-dsDNA) antibodies, prednisone dose, and SLE Disease Activity Index 2000 (SLEDAI-2K) scores, were analyzed using mixed-effects modeling (REML). Correlations between changes in clinical SLEDAI-2K (excluding serological components) and serological markers were assessed.

**Results:**

Thirteen patients (84.6% female, median age 53 years) were included. The median baseline SLEDAI-2K was 10, and 76.9% exhibited abnormal complement and/or anti-dsDNA levels. Over the treatment course (median 12 infusions), 76.9% of patients improved clinically, with a mean SLEDAI-2K reduction of 3.77 ± 2.78 points (p < 0.001). Prednisone doses decreased in 38.5% of cases. Complement (C3c, p = 0.25; C4, p = 0.10) and anti-dsDNA levels (p = 0.12) remained largely unchanged. No correlations were observed between clinical SLEDAI-2K improvement and serological parameters.

**Discussion:**

Anifrolumab therapy led to significant clinical improvement without corresponding serological changes, suggesting that traditional biomarkers may not adequately reflect therapeutic response. Monitoring under ANI should therefore emphasize clinical rather than serological parameters. These findings have implications for interpreting composite disease activity indices incorporating immunological markers in SLE management depending on the mechanism of action of a particular treatment.

## 1 Introduction

Anifrolumab (ANI) is a monoclonal antibody approved for systemic lupus erythematosus (SLE) based on its clinical benefits shown in the TULIP trials (1, 2). However, its influence on serological markers, commonly used in clinical practice to guide the therapeutic response, remains uncertain. Since validated disease activity indices, such as the Systemic Lupus erythematosus Disease Activity Index (SLEDAI) (3), are not widely used outside trials (4), we aimed to clarify how ANI affects key serological parameters in routine care. We, therefore, retrospectively analyzed changes in serological markers among SLE patients treated with ANI since January 2022 at a single center.

## 2 Methods

### 2.1 Patient cohort and data collection

This was a single-center retrospective analysis conducted at the Department of Nephrology and Rheumatology, University Medical Center Göttingen, Germany. We only included patients who had received ≥3 infusions during an observation period of up to 12 months.

Eligible patients were adults (≥18 years) with a confirmed diagnosis of SLE according to the 2019 EULAR/ACR classification criteria or the 1997 ACR revised criteria. Inclusion required treatment with ANI (300 mg intravenously every 4 weeks) with a minimum of three infusions administered. Patients who discontinued therapy before the third infusion were excluded. There was no restriction on concomitant immunomodulatory therapy. Patients were identified through the institutional pharmacy records for ANI dispensing between January 2022 and March 2024.

We collected demographic and clinical data, along with complement factors (C3c, C4), anti-dsDNA antibody levels, disease activity using the SLEDAI-2K, and daily prednisone dose. Demographic variables included age, sex, and disease duration. Clinical data encompassed SLE manifestations classified by SLEDAI-2K organ domains, concomitant and prior immunomodulatory therapies (hydroxychloroquine, azathioprine, mycophenolate mofetil, methotrexate, cyclophosphamide, belimumab, and rituximab), and the number of ANI infusions received. C3c and C4 were determined by nephelometry, and anti-dsDNA antibodies by ELISA. Serological parameters and clinical assessments were obtained at each routine clinical visit every 4 weeks. Accordingly, data were collected at baseline (prior to the first infusion) and at infusion visits 2 – 12, as applicable. Of note, not every patient had complete serological data at each visit.

### 2.2 Statistical analysis

We analyzed changes in the SLEDAI-2K and prednisone doses before and after the use of ANI. Data with repeated measures were analyzed using a mixed-effects model (restricted maximum likelihood, REML). This method accounts for repeated measures and can handle missing values without the need for imputation.

Further, we performed a correlation analysis to assess the correlation of the clinical SLEDAI-2K with serological parameters. For this analysis, we recorded the clinical SLEDAI-2K (SLEDAI-2K without complement and anti-dsDNA levels) at baseline and at last follow-up. Spearman rank correlation coefficients were calculated to assess the association between the change in clinical SLEDAI-2K (ΔcSLEDAI-2K) and the change in each serological parameter (ΔC3c, ΔC4, Δanti-dsDNA) from baseline to last follow-up. 95% confidence intervals for the correlation coefficients were computed.

All analyses were performed with GraphPad Prism version 10.4.2 for Mac, GraphPad Software, Boston, Massachusetts USA, www.graphpad.com. A two-sided p-value < 0.05 was considered statistically significant.

## 3 Results

### 3.1 Baseline characteristics

In our cohort, there were 13 (11 female, 84.6%) patients with sufficient clinical data. Baseline characteristics are displayed in **Table 1**. The median age was 53 (31–78) years, the median disease duration was 10 (2–18) years. All patients were positive for antinuclear antibodies (ANA). Ten of 13 patients (76.9%) were positive for anti-dsDNA antibodies at any time during their disease course. Additional autoantibody specificities included anti-Sm (n = 3), anti-SSA (n = 5), anti-SSB (n = 3), anti-U1RNP (n = 4), anti-histone (n = 2), anti-ribosomal P (n = 1), and anti-Mi-alpha (n = 1). The most frequent concomitant immunomodulatory therapies at baseline were hydroxychloroquine (n = 9, 69.2%), azathioprine (n = 6, 46.2%), and mycophenolate mofetil (n = 3, 23.1%). Five patients (38.5%) had previously received belimumab, and three (23.1%) had previously received rituximab. At baseline, the median SLEDAI-2K score was 10 (8–24), and 10 of 13 (76.9%) patients had decreased complement levels and/or increased anti-double stranded (ds) DNA levels. The most common clinical SLEDAI-2K domains at baseline were arthritis (n = 7, 53.8%), rash (n = 6, 46.2%), neuropsychiatric SLE (NPSLE, n = 4, 30.8%), mucosal ulcers (n = 3, 23.1%), and hematuria (n = 2, 15.4%). Individual patient-level data including demographics, autoantibody profiles, concomitant therapies, and SLEDAI-2K domain involvement are presented in the **Supplementary Table**.

**Table 1:** Characteristics of SLE patients treated with anifrolumab.

No.	Age	Sex	Dis. Dur. (y)	anti-dsDNA	Other Ab	Conc. Rx	ANI Inf.	Prednisolon (mg/d)	SLEDAI BSL	ΔSLEDAI (last follow up)
1	34	M	3	+	Sm, SSA, U1RNP	–	11	7.5 → 20	20	-4
2	31	F	15	+	Sm, SSA, U1RNP, Histone	HCQ, AZA	12	5 → 0	10	-6
3	78	M	–	–	Sm, U1RNP	HCQ	12	0 → 0	10	-8
4	63	F	2	+	SSA, SSB, Ro52, KU, CCP, RF	HCQ, AZA	3	0 → 0	8	-4
5	33	F	11	–	–	HCQ, MMF	12	10 → 7	24	-4
6	58	F	2	+	–	HCQ, AZA	3	1.25 → 0	10	-8
7	41	F	7	+	RibP	HCQ	3	5 → 0	12	-4
8	53	F	5	+	–	HCQ, MTX	7	5 → 0	14	-6
9	61	F	18	+	Mi-Alpha	–	9	0 → 0	10	0
10	38	F	18	+	SSA, SSB	HCQ	3	5 → 0	14	-2
11	65	F	11	+	SSA, SSB	AZA	12	5 → 5	9	-3
12	38	F	10	–	SSA, U1RNP	AZA, MMF	3	7 → 0	14	0
13	62	F	–	+	Histone	HCQ, AZA	12	5 → 7.5	8	-4

ANI Inf., number of anifrolumab infusions; AZA, azathioprine; BSL, baseline; Conc. Rx, concomitant therapy at baseline; Dis. Dur., disease duration; anti-dsDNA, double-stranded DNA antibodies; HCQ, hydroxychloroquine; MMF, mycophenolate mofetil; MTX, methotrexate; Other Ab, other autoantibodies; SLEDAI, Systemic Lupus erythematosus Disease Activity Index; y, year.

The median number of infusions in one year was 9 (range 3–12), the duration of follow-up was 12 months on an individual patient level.

### 3.2 Clinical response

**Figure 1:**
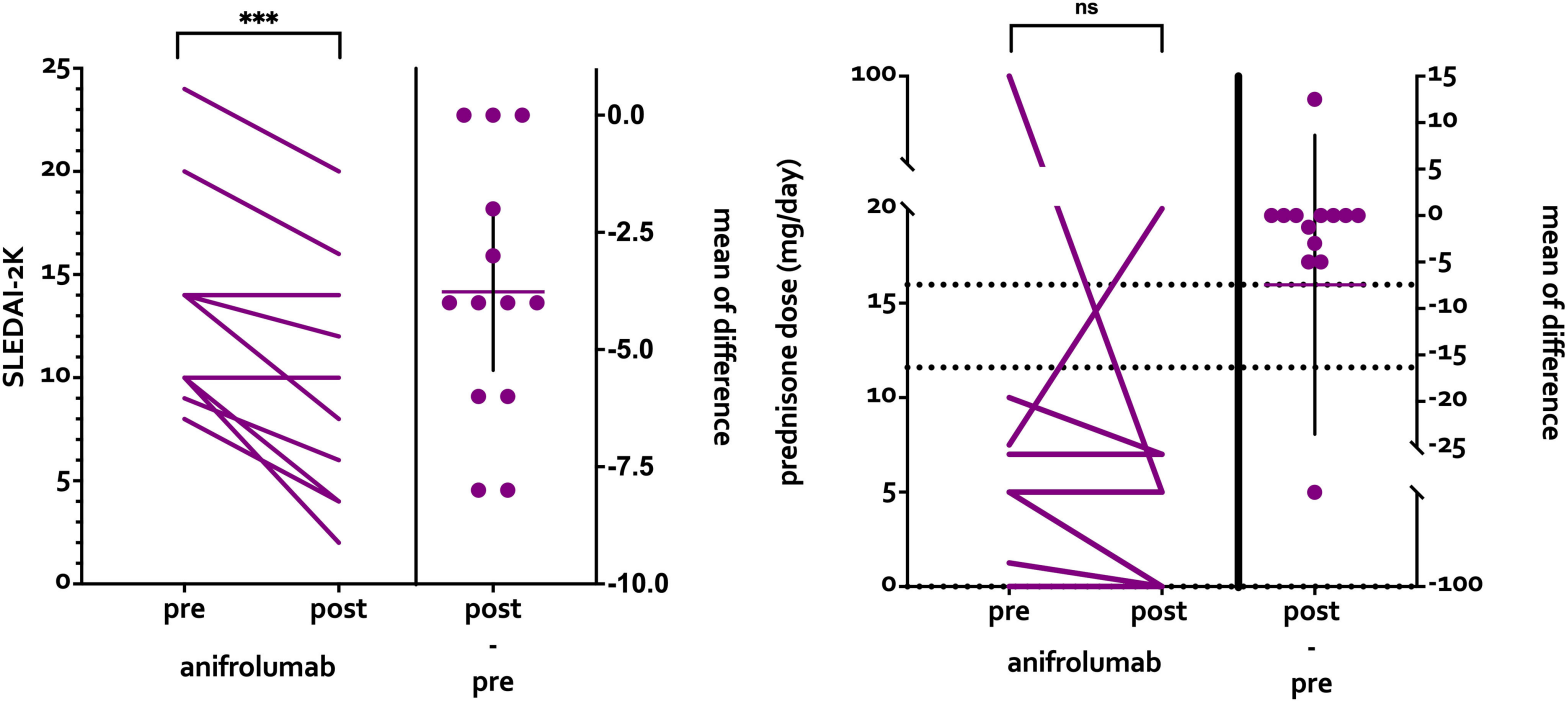
SLEDAI-2k and prednisone dose before and after anifrolumab therapy.

During follow-up, 10/13 (76.9%) patients showed clinical improvement, the SLEDAI-2K decreased by 3.77 ± 2.78 points (p<0.001). A prednisone reduction was achieved in 5/13 (38.5%) patients, which was not statistically significant (**Figure 1****).** Specifically, of the five patients with prednisone reduction, four achieved complete discontinuation (from a median of 5 mg/day to 0 mg/day). Two patients (15.4%) had an increase in prednisone dose during follow-up (patient 1: from 7.5 to 20 mg/day; patient 13: from 5 to 7.5 mg/day).

### 3.3 Serological parameters

**Figure 2:**
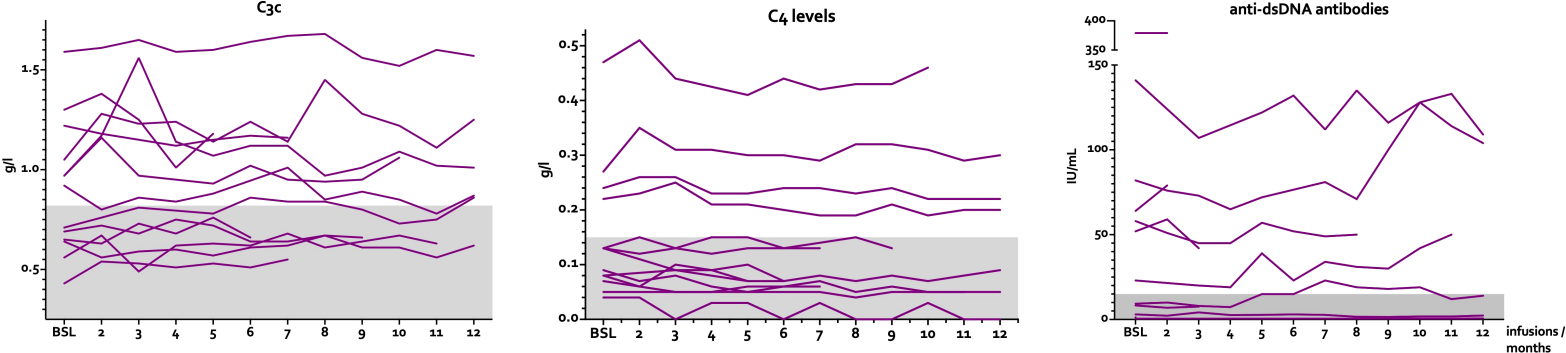
Levels of C3c, C4, and double-stranded DNA antibodies over time.

Using a mixed-effects model for repeated measures, complement and anti-dsDNA levels remained largely unchanged during follow-up (**Figure 2**). There were no statistically significant changes during the course of the treatment (C3c, p=0.2502; C4, p=0.1013; anti-dsDNA, p=0.1164). At baseline, median C3c was 0.92 g/L (0.43-1.59), median C4 was 0.13 g/L (0.04-0.47), and median anti-dsDNA was 37.5 IU/mL (0.5-379]). At last follow-up, median C3c was 0.87 g/L (0.55-1.57), C4 was 0.09 g/L (0-0.46), and anti-dsDNA was 14 IU/mL (0.6-109). The mean absolute changes from baseline to last follow-up were ΔC3c = 0.06 g/L (0.01-0.2), ΔC4 = 0.02 g/L (0-0.06), and Δanti-dsDNA = 1.8 IU/mL (0-32).

### 3.4 Correlation between clinical and serological changes

Further, we analyzed whether the observed differences of the clinical SLEDAI-2K before and after treatment (ΔcSLEDAI-2K) correlated with changes of the complement or anti-dsDNA levels. Here, we observed no correlation between changes in cSLEDAI-2K scores and serological parameters (**Table 2**). The Spearman correlation coefficients were as follows: ΔcSLEDAI-2K *vs*. C3c: r = −0.40 (95% CI −0.79 to 0.21, p = 0.17), ΔcSLEDAI-2K *vs*. C4: r = 0.22 (95% CI −0.39 to 0.70, p = 0.46), and ΔcSLEDAI-2K *vs*. anti-dsDNA: r = −0.27 (95% CI −0.70 to 0.34, p = 0.36). These findings indicate that clinical improvement under ANI therapy occurs independently of changes in anti-dsDNA and complement levels.

**Table 2:** Spearman correlation coefficients between clinical SLEDAI and C3c, C4, and double-stranded DNA antibodies.

Δ cSLEDAI *vs*.	C3c	C4	dsDNA
Spearman r	-0.40 (CI -0.79 – 0.21)	0.22 (CI -0.39 – 0.70)	-0.27 (CI -0.7 – 0.34)
p-value	0.17	0.46	0.36

cSLEDAI, clinical Systemic Lupus erythematosus Disease Activity Index.

## 4 Discussion

Our results reaffirm ANI’s effectiveness in clinical practice but show that relying solely on serological parameters may be insufficient for monitoring response. The dissociation between clinical and serological outcomes suggests that clinicians should prioritize clinical domains, especially since SLEDAI-2K may not reflect improvement under ANI. Thus, assessment should emphasize clinical findings rather than complement or antibody levels alone. The observed discordance between clinical improvement and serological stability can be understood through the mechanism of action of anifrolumab. By blocking IFNAR1, ANI suppresses type I IFN-driven inflammation, which manifests clinically through improvements in skin disease, arthritis, and other organ involvement (1, 2). However, autoantibody production and complement consumption in SLE are driven primarily by B-cell hyperactivation and immune complex formation, pathways that are only partially dependent on type I IFN signaling (5). While type I IFN can promote B-cell differentiation and BAFF expression, the established pool of long-lived plasma cells producing anti-dsDNA antibodies may be relatively resistant to IFN blockade alone (6). Similarly, complement activation through the classical pathway is driven by circulating immune complexes and may persist even when clinical disease activity improves through IFN-independent mechanisms.

Our findings are consistent with the TULIP trial data. In the TULIP-2 trial, ANI achieved significantly higher BICLA response rates compared to placebo (47.8% *vs*. 31.5%), yet changes in complement and anti-dsDNA levels were modest (1). *Post-hoc* analyses of pooled TULIP data demonstrated that patients with low complement and/or elevated anti-dsDNA at baseline showed numerically greater clinical responses (7). However, only a minority of patients with elevated anti-dsDNA achieved seronegativity during the trial (8). These trial data support our real-world observation that ANI’s clinical efficacy is not primarily mediated through serological normalization which stands in marked contrast to other biologic therapies used in SLE. Belimumab (BEL), which directly inhibits BAFF and thereby reduces B-cell survival and autoantibody production, has been shown to reduce anti-dsDNA levels by approximately 41% and to normalize complement (C3 and C4) over 52 weeks (9). Similarly, the CD20-mediated B-cell depleting monoclonal antibody rituximab (RTX) achieves significant reductions in anti-dsDNA antibodies and normalization of complement levels in the majority of treated patients (10). These differences underscore the mechanistic heterogenicity of biologic therapies in SLE and highlight that serological response depends on the therapeutic target: agents directly targeting B cells (BEL, RTX) normalize serological markers, whereas ANI, targeting upstream innate immunity through IFN blockade, achieves clinical improvement predominantly through IFN-dependent pathways.

Given the dissociation between clinical and serological outcomes, alternative pharmacodynamic biomarkers should be considered for monitoring ANI therapy. The type I IFN gene signature (IFNGS) provides more objective evidence of IFNAR1 blockade (11, 12). However, IFNGS measurement is not routinely available in clinical practice. Other potential biomarkers under investigation include downstream transcriptomic changes, plasma proteomic markers (e.g., CXCL10/IP-10, TRAIL, MCP-1), and changes in B-cell subsets, particularly follicular helper T cells (13). Future studies should evaluate the clinical utility and feasibility of these biomarkers for monitoring ANI therapy.

Although limited by a small sample size and retrospective design, our findings provide insights for interpreting serological markers during ANI therapy. Clinical assessment should guide disease activity evaluation, as serological markers may lack relevance depending on the mechanism of action. This is important when using composite indices, like SLEDAI-2K, which incorporate serological parameters. In clinical practice, physicians often rely on changes of serological responses to judge the efficacy of any treatment in SLE. However, our data demonstrates that this assumption is incorrect with ANI, which can be explained by its mechanism of action.

## 5 Conclusions

In conclusion, the assessment of ANI’s therapeutic efficacy should rely on validated disease indices, such as the clinical SLEDAI-2K, rather than changes of serological parameters alone. In addition, the SLEDAI-2K incorporating immunological parameters may be misleading. To our knowledge, this is the first specific analysis of serological parameters and clinical responses in a cohort of SLE patients treated with ANI.

## Data Availability

The original contributions presented in the study are included in the article/**Supplementary Material**. Further inquiries can be directed to the corresponding author.

## Supplementary material

Supplementary Table SLEDAI-2K domain involvement at baseline and follow-up.
