# Supplementary material for: Complement and autoantibody levels under anifrolumab therapy in SLE: implications for clinical practice
Source: Front Immunol. 2026 Mar 11;17:1737281. doi: 10.3389/fimmu.2026.1737281 (PMC13013301; doi:10.3389/fimmu.2026.1737281)
Supplement: Supplementary Table — SLEDAI-2K domain involvement at baseline and follow-up. [file Table1.docx]

Complement and Autoantibody Levels under Anifrolumab Therapy in SLE: Implications for Clinical Practice

Jan-Gerd Rademacher^1^, Björn Tampe^1^, Peter Korsten^1,2*^

***Supplementary Table: SLEDAI-2K Domain Involvement at Baseline and Follow-up***

| Pt. | SLEDAI-2K Domains at Baseline | SLEDAI BSL | SLEDAI-2K Domains at Follow-up | SLEDAI FU |
| --- | --- | --- | --- | --- |
| 1 | NPSLE, Myositis, Fever, Rash, Low C, High dsDNA, Leukopenia | 20 | NPSLE, Myositis, Low C, High dsDNA | 16 |
| 2 | Arthritis, Rash, Low C, High dsDNA | 10 | Low C, High dsDNA | 4 |
| 3 | Vasculitis, Low C | 10 | Low C | 2 |
| 4 | Pyuria, Low C, High dsDNA | 8 | Low C, High dsDNA | 4 |
| 5 | NPSLE, Arthritis, Mucosal ulcers, Rash | 24 | NPSLE, Arthritis | 20 |
| 6 | Arthritis, Rash, Pericarditis, Low C | 10 | Low C | 2 |
| 7 | Arthritis, Hematuria, Low C, High dsDNA | 12 | Arthritis, Low C, High dsDNA | 8 |
| 8 | NPSLE, Arthritis, Mucosal ulcers | 14 | NPSLE | 8 |
| 9 | Arthritis, Myositis, High dsDNA | 10 | Arthritis, Myositis, High dsDNA | 10 |
| 10 | Arthritis, Rash, Hematuria, Low C, High dsDNA | 14 | Arthritis, Hematuria, Low C, High dsDNA | 12 |
| 11 | Alopecia, Rash, Pyuria, Leukopenia | 9 | Pyuria, High dsDNA | 6 |
| 12 | NPSLE, Mucosal ulcers, Rash, Low C | 14 | NPSLE, Mucosal ulcers, Rash, Low C | 14 |
| 13 | Rash, Pleuritis, Low C, High dsDNA | 8 | Low C, High dsDNA | 4 |

BSL, baseline; FU, follow-up; Pt., patient; SLEDAI, Systemic Lupus erythematosus Disease Activity Index
